# Adherence to Healthy Dietary Patterns and Cognitive Performance: Evidence from DASH and Mediterranean Diets

**DOI:** 10.3390/nu18111702

**Published:** 2026-05-27

**Authors:** Seher Abasız, Müge Arslan

**Affiliations:** 1 Nutrition and Dietetics, Institute of Health Sciences, Uskudar University, Istanbul 34662, Turkey; 2 Nutrition and Dietetics, Faculty of Health Sciences, Uskudar University, Istanbul 34662, Turkey; muge.arslan@uskudar.edu.tr

**Keywords:** Mediterranean diet, DASH diet, dietary patterns, cognition, memory, executive function, adult

## Abstract

**Background/Objectives**: This study aimed to evaluate the effect of adherence to the DASH and Mediterranean diets on cognitive performance in adults. **Methods**: In this study, the Mediterranean Diet Adherence Screener (MEDAS), DASH Diet Quality Scale (DASH-Q), Oktem Verbal Memory Processes Test (Oktem-VMPT), and Trail Making Test (TMT) were administered face-to-face to adult individuals living in Afyonkarahisar, Türkiye, together with a form assessing sociodemographic characteristics and dietary habits. The collected data were analyzed using SPSS v27 software. **Results**: As participants’ ages increased, DASH scores decreased (*p* < 0.05). As participants’ BMI and waist/hip width increased, a decrease in DASH and MEDAS scores was observed (*p* < 0.05). As participants’ ages increased, the IST-A, IST-B, and IST-Total scores increased (*p* < 0.05), but as their education level increased, the IST-A, IST-B, and IST-Total scores decreased (*p* < 0.05). As participants’ education level increased, the total recall score on the Oktem-SBST scale tended to increase (*p* < 0.05). As participants’ DASH scores increased, the “Immediate Memory” and “Spontaneous Recall” sub-components of the Oktem-SBST increased, while the “Learning Mistake Score,” “USB Mistake Score,” and “IST-A,” “IST-B,” and “IST Total” scores decreased (*p* < 0.05). As participants’ MEDAS scores increased, the sub-components of Oktem-SBST, namely “Criteria Achievement,” “Maximum Learning,” “Spontaneous Recall,” “Recognition,” and “Total Recall,” also increased, while the “Learning Mistake Score” decreased. (*p* < 0.05). **Conclusions**: Age, educational status, DASH, and MEDAS scores are associated with cognitive performance. The DASH and MEDAS diets have a positive impact on cognitive performance, highlighting the importance of healthy eating in public health strategies for maintaining cognitive health.

## 1. Introduction

Cognitive capacity is a concept encompassing the mental processes of the human mind, such as reasoning, memory, perception, learning, comprehension, concentration, decision-making, intuitive thinking, and language skills [1]. Mental performance can be influenced by various factors, including dietary habits, sleep patterns, age, smoking and alcohol consumption, environmental factors, exercise, and sports [2]. Among these factors, nutrition holds a particularly important place, and unbalanced nutrition and deficiencies in certain essential trace elements may contribute to the development of psychological and neurological diseases such as depression and dementia by leading to impaired brain functions [3]. Daily dietary habits have a determining effect on cognitive function in both the short and long term through central and peripheral mechanisms. While a balanced diet protects and supports brain structure and functions, inadequate nutrition may disrupt this balance and pose a risk. Improving dietary patterns may be an important strategy for strengthening mental health and slowing age-related cognitive decline [4,5].

Research has shown that high adherence to the Mediterranean diet may reduce the risk of cognitive impairment and Alzheimer’s disease [6,7]. The Mediterranean diet is a dietary pattern based on the high consumption of locally sourced and minimally processed fruits and vegetables, as well as nuts, legumes, whole grains, seeds, and olive oil as the main source of fat [8]. Adherence to this dietary pattern has been associated with improvements in overall cognitive functions and episodic memory performance, reductions in the risk of cognitive impairment and neurodegenerative diseases, and a slowing of the rate of cognitive decline associated with Alzheimer’s disease [9]. Studies have shown that, in older adults who adopt the Mediterranean diet, overall cognitive performance is higher, the decline in mental abilities progresses more slowly, the risk of dementia is reduced, and life expectancy is prolonged [10,11]. In a study conducted in the United States on individuals aged 55 years and older, it was reported that individuals with high adherence to the Mediterranean diet performed better in terms of attention, information processing speed, and higher-order cognitive functions [12].

Studies have also shown that the DASH diet is effective on cognitive functions [13,14]. The DASH diet is a dietary pattern that recommends the consumption of fruits and vegetables as well as foods rich in potassium, magnesium, calcium, and fiber; it also encourages the intake of low-fat dairy products, poultry, whole grains, seafood, and nuts, while recommending the limitation of sodium, saturated fat, and total fat consumption [15]. Better cognitive performance has been identified in individuals who adopt the DASH dietary pattern [16]. In a study conducted by Daniel et al. with 4169 participants, the association between adherence to the DASH diet and cognitive function was examined, and it was concluded that adherence to the DASH diet was not associated with any adverse association with cognitive performance or related measures [14]. Similarly, a study conducted by Key and Szabo-Reed examined the effects of diet and exercise interventions on cognitive functions and brain health and emphasized that the DASH diet may provide significant benefits in these areas [17].

This study is an original study that fills the gap in the literature by evaluating the combined use of both DASH and Mediterranean dietary patterns together with the Oktem-VMPT and TMT, and it may serve as a guide for future studies on this subject.

## 2. Materials and Methods

### 2.1. Study Design, Participants, and Variables

This study was based on a correlational survey model and had a cross-sectional design. It was conducted among adults aged 18–65 years living in Afyonkarahisar between September 2024 and December 2025, after receiving ethical approval from the Non-Interventional Research Ethics Committee of Üsküdar University at its Meeting No. 12 held on 29 December 2024.

The study population consisted of adult individuals aged 18–65 years living in Afyonkarahisar. Although the use of physician-prescribed medications was listed as an exclusion criterion, 26 participants (4.3%) reported regular medication use at the time of data collection. These participants were retained in the analysis because their medications were not considered likely to confound dietary assessment or cognitive performance outcomes. To verify that their inclusion did not affect the results, a sensitivity analysis was conducted by comparing DASH and MEDAS scores between participants who used medication regularly and those who did not; no statistically significant difference was found (DASH: *p* = 0.988; MEDAS: *p* = 0.415). These findings suggest that the inclusion of these participants did not meaningfully influence the study outcomes.

Participants were recruited through face-to-face invitations at community centers, public spaces, and university campus areas in Afyonkarahisar. Approximately 700–750 individuals were approached, of whom 600 agreed to participate. Between 100 and 150 individuals declined to participate, primarily citing time constraints and unwillingness to complete the lengthy questionnaire battery. Participation was voluntary and non-consecutive.

Inclusion criteria were being aged between 18 and 65 years, living in Afyonkarahisar, not being pregnant or breastfeeding, not having congenital or acquired conditions affecting eating behavior (such as cleft palate, tongue, or dental problems), not having any physician-diagnosed psychiatric disorder, not using physician-prescribed medications, and not having physical, mental, or cognitive impairments. Exclusion criteria were being younger than 18 years of age and not living in Afyonkarahisar.

The independent variables of the study were the Mediterranean Diet Adherence Scale scores and DASH Diet Quality Scale scores, whereas the dependent variables were the Oktem Verbal Memory Processes Test scores and Trail Making Test scores.

### 2.2. Data Collection Tools

In this study, the Mediterranean Diet Adherence Screener (MEDAS), DASH Diet Quality Scale (DASH-Q), Oktem Verbal Memory Processes Test (Oktem-VMPT), and Trail Making Test (TMT) were administered face-to-face using pen-and-paper forms by the researchers to adult individuals living in Afyonkarahisar, Türkiye, together with a form assessing sociodemographic characteristics and dietary habits. The collected data were analyzed using SPSS (IBM Corp., Armonk, NY, USA) v27 software.

#### 2.2.1. Sociodemographic Data Form

Data on age, sex, marital status, educational level, occupation, anthropometric measurements (body weight, height, BMI, waist and hip circumferences, and waist-to-hip ratio), the presence of any physician-diagnosed chronic disease, physician-prescribed medications or dietary supplements used, alcohol and smoking habits and, if applicable, their amounts, meal patterns including which meals were skipped, and daily water consumption were obtained from the adult individuals participating in the study. Anthropometric measurements were taken by the researcher in a clinical setting using a TANITA body composition analyzer for body weight and BMI, and a standard measuring tape for waist and hip circumferences, following standard protocols. The presence of chronic disease and medication use was determined based on self-report by the participants.

#### 2.2.2. Oktem Verbal Memory Processes Test (Oktem-VMPT)

This test was designed to measure verbal learning processes and memory functions in a detailed and multidimensional manner. Within the scope of the test, a list consisting of 15 words is presented to the participant in a neutral voice, with a 1-s interval between each word. After each trial, the participant is asked to recall and state the words in any order. The first stage of the test ends when all words are recalled or when 10 trials have been completed. In the second stage, long-term memory (LTM) is evaluated, and the delayed recall is assessed approximately 30 min after the learning phase (maximum interval: 30 min), during which the participant is asked to recall and repeat the word list. The words that cannot be recalled are identified by selecting them from a recognition list. At the end of the test, depending on the participant’s performance, the scores for total learning, immediate memory, perseveration, learning error, inconsistency, reaching criterion, and highest learning related to “learning processes” are calculated in the first stage. In the second stage, the scores for LTM recall error, spontaneous recall, total recall, recognition, and false recognition related to “long-term memory” are evaluated [18].

#### 2.2.3. Trail Making Test (TMT)

The Trail Making Test was developed in 1944 as part of the Army Individual Test Battery and was structured to consist of two separate forms, referred to as Forms A and B. Form A includes a task requiring the ordering of numbers presented in a scattered manner from 1 to 25, whereas Form B follows a sequence that must be arranged in the form of one number and one letter. Form B includes the numbers from 1 to 13 and the letters from A to L. The test is administered under timed conditions; the time limit is 180 s for Form A and 300 s for Form B. The TMT score was based on the time taken to complete each form, recorded in seconds. If errors were made, participants were immediately corrected and asked to continue without stopping the clock. As age increases, an increase is observed in the completion time of both forms. The Trail Making Test is an assessment tool requiring detailed visual scanning, and the adaptation and standardization study of the scale for Turkish society was conducted on a sample consisting of individuals aged 50 years and older [19]. Owing to its ability to measure higher-order executive functions such as complex attention processes, response inhibition, planning skills, and cognitive flexibility, this test is among the tools commonly preferred in clinical neuropsychometric evaluations [19,20].

#### 2.2.4. DASH Diet Quality Scale (DASH-Q)

Within the scope of this study, the DASH Diet Quality Scale (DASH-Q) was used to determine the participants’ level of adherence to the DASH diet. This scale was developed by Warren-Findlow et al. to evaluate adherence to the DASH diet in adults [21], and its validity and reliability analyses for Turkish society were conducted by Çetin (2020) [22].

This 11-item scale questions how frequently the participants consumed certain foods and food groups during the last 7 days. Therefore, the DASH-Q reflects recent dietary behavior rather than long-term habitual dietary intake. All questions were prepared in the format of “During the last 7 days, on how many days did you consume ………?”. If the specified food group was not consumed at all, 0 points are assigned, whereas if it was consumed every day, 7 points are assigned; thus, each item is scored between 0 and 7.

The total scores obtained are evaluated as follows:

- 0–32 points: low diet quality,
- 33–51 points: moderate diet quality,
- 52–77 points: high diet quality (high adherence to the DASH diet).

#### 2.2.5. Mediterranean Diet Adherence Scale (MEDAS)

In this study, the Mediterranean Diet Adherence Scale (MEDAS) was used to evaluate the participants’ level of adherence to the Mediterranean-style dietary pattern. This 14-item scale was developed by Martínez-González et al. (2012) [23]. The validity and reliability analyses of its Turkish adaptation were conducted by Pehlivanoğlu et al. (2020) [24].

Each item is scored as 1 or 0 based on the amount consumed, and the total score is calculated accordingly. The total score obtained is classified as follows:

- 0–6 points: low adherence to the Mediterranean diet,
- 7–8 points: acceptable adherence to the Mediterranean diet,
- 9 points and above: high adherence to the Mediterranean diet.

This scale was used to reliably measure the participants’ Mediterranean-style dietary habits.

### 2.3. Statistical Analysis

In sample size calculation, in addition to the calculation method (d) developed by Cohen, different effect size measures such as Hedge’s d and Glass’s Δ are also frequently included in the literature. According to Cohen’s general classification, a d value below 0.2 indicates a small effect size, a value around 0.5 indicates a medium effect size, and a value above 0.8 indicates a large effect size. However, it should not be overlooked that even a small d value such as 0.2 may be considered an important effect in some specific situations [25,26]. Within the scope of the study, Cohen’s effect size was determined as r = 0.275 [20]. Power analysis was performed using R v4.3.1 software; the alpha error level was accepted as 5% and the beta error level as 20%. Assuming that there would be a significant difference between the variables, the required minimum sample size was determined as 181 [27,28]. Considering the accessibility of participants and the possibility of incomplete responses, the sample size was increased to 600 individuals. In addition, a larger sample size was targeted to improve statistical power, increase the representativeness of the study population, and allow more reliable subgroup evaluations [29].

Categorical variables were summarized using frequency and percentage values. The distribution characteristics of continuous variables were evaluated using the Shapiro–Wilk test. Data meeting the assumption of normal distribution were expressed as mean and standard deviation (mean ± SD), whereas data not meeting this assumption were expressed using median and minimum–maximum values.

For data that did not meet the assumption of normal distribution, the Mann–Whitney U test was used for comparisons between two independent groups. For comparisons among three or more independent groups, the Kruskal–Wallis H test was preferred. The results of multiple comparisons were presented using the letter-based grouping method by taking median values into consideration.

The relationships between numerical variables that did not show normal distribution were examined using Spearman’s rank-order correlation coefficient. According to the magnitude of the correlation coefficient, the level of relationship was evaluated as very weak for values below 0.20, weak for values between 0.20 and 0.40, moderate for values between 0.40 and 0.60, strong for values between 0.60 and 0.80, and very strong for values above 0.80 [30]. A Bonferroni correction was applied to control for the possibility of Type I errors arising from multiple comparisons, and the significance level was set at *p* < 0.002.

Spearman’s rank-order correlation was used to examine bivariate associations between DASH and MEDAS scores and continuous variables (age, BMI, waist-to-hip ratio) and Oktem-VMPT and TMT subcomponents, without controlling for other factors. Multiple linear regression was subsequently used to assess the independent predictive effects of DASH and MEDAS scores on cognitive outcomes while simultaneously adjusting for potential confounding variables including age, sex, educational status, and income level. Although some variables did not meet the assumption of normal distribution, multiple linear regression was considered appropriate given the large sample size (*n* = 600), as the method is robust to normality violations under such conditions. A ‘multiple linear regression’ analysis was conducted to examine the predictive effects on the dependent variables. To assess the independent variables in the model for multicollinearity, the variance inflation factor (VIF) and tolerance values were examined. As the VIF values were below 5 and the tolerance values were above 0.20, it was determined that there was no multicollinearity.

Residual analyses were conducted to assess whether the assumptions of the regression model were met. In this context, standardized residuals and Cook’s Distance were examined. It was observed that the standardized residuals remained within acceptable limits of ±3 and that the Cook’s Distance values were below 1. Based on these findings, it was concluded that the fundamental assumptions of the regression models were met.

In the analyses performed in the study, the level of significance was accepted as *p* < 0.05, *p* < 0.01, and *p* < 0.001, and all tests were conducted according to the two-tailed hypothesis approach. Statistical evaluations were performed using IBM SPSS Statistics 27 (IBM Corp., Armonk, NY, USA).

## 3. Results

Of the participants, 42.8% were men and 57.2% were women. Among men, 67.7% were married, 50.2% had a university level of education, 53.3% had an income greater than their expenses, 34.2% smoked, 5.8% consumed alcohol, 12.1% had a chronic disease, 3.5% used medication regularly, 11.3% used dietary supplements regularly, 58.4% skipped meals, and 48.6% consumed 1.5–2 L/day of water. Among women, 51.9% were single, 53.9% had a university level of education, 47.2% had an income lower than their expenses, 11.7% smoked, 2.9% consumed alcohol, 14.6% had a chronic disease, 5.0% used medication regularly, 13.7% used dietary supplements regularly, 75.5% skipped meals, and 45.2% consumed 1.5–2 L/day of water (Table 1).

Of the participants, 56.5% were married, 52.3% had a university level of education, 42.0% had an income greater than their expenses, 21.3% smoked, 4.2% consumed alcohol, 13.5% had a chronic disease, 4.3% used medication regularly, 12.7% used dietary supplements regularly, 68.2% skipped meals, and 46.7% consumed 1.5–2 L/day of water (Table 1).

The mean age of the participants was 36.04 ± 12.91 years, and the mean BMI was 25.35 ± 3.86. The mean total MEDAS score was 7.43 ± 1.87, whereas the mean total DASH score was 36.27 ± 6.91. When the Oktem-VMPT subcomponents were examined, the mean scores were determined as 5.57 ± 1.21 for “Immediate Memory,” 107.54 ± 18.38 for “Learning Score,” 13.62 ± 1.98 for “Reaching Criterion,” 13.62 ± 1.98 for “Highest Learning,” 0.86 ± 0.82 for “Learning Error Score,” 12.14 ± 2.15 for “Spontaneous Recall,” 13.69 ± 1.95 for “Recognition,” and 25.82 ± 4.00 for “Total Recall.” The mean “LTM Error Score” was 0.24 ± 0.49. The mean TMT scores were 32.43 ± 9.32 for “TMT-A,” 69.73 ± 20.24 for “TMT-B,” and 102.16 ± 29.38 for “Total TMT” (Table 2).

In the analysis performed according to sex, significant differences were found in the participants’ age, BMI, waist-to-hip ratio, MEDAS, and DASH scores, as well as in the Oktem-VMPT subcomponent scores for “Immediate Memory,” “Learning Score,” “Reaching Criterion,” “Highest Learning,” “Learning Error Score,” “Spontaneous Recall,” “Recognition,” and “Total Recall,” and in the TMT subcomponent scores for TMT-A and TMT-B, and in the Total TMT score (*p* < 0.05; *p* < 0.01; *p* < 0.001) (Table 2).

Statistically significant differences were observed in the participants’ DASH and MEDAS scores according to age groups. For DASH, the median values were higher in individuals aged 18–35 years [37 (17–68)] and 36–50 years [36 (23–54)] than in those aged 51–65 years [33.5 (23–47)], and similarly, for MEDAS, the median value was higher in individuals aged 18–35 years [8 (3–13)] than in those aged 36–50 years [7 (4–11)] and 51–65 years [7 (3–12)] (Table 3).

A statistically significant, negative, and very weak relationship was found between participants’ age and DASH scores (*p* < 0.01). As age increased, DASH scores tended to decrease (Table 3).

Statistically significant differences were observed in the participants’ DASH and MEDAS scores according to marital status. For DASH, the median value was higher in single individuals [37 (17–61)] than in married individuals [35 (21–68)], and similarly, for MEDAS, the median value was also higher in single individuals [8 (3–12)] than in married individuals [7 (3–13)] (Table 3).

Statistically significant differences were observed in the participants’ DASH and MEDAS scores according to educational status. For DASH, the median values were higher in individuals with a postgraduate education level [38 (17–59)] and in individuals with a university education level [37 (21–68)] than in individuals with a high school education level [35 (24–50)] and in literate individuals [32 (24–53)]; the median value of individuals with a high school education level [35 (24–50)] was also higher than that of literate individuals [32 (24–53)]. Similarly, for MEDAS, the median value of individuals with a postgraduate education level [8 (5–13)] was higher than that of individuals with a university education level [7 (3–12)], a high school education level [7 (3–12)], and a primary education level [7 (3–10)]; the median value of literate individuals [8 (4–12)] was also higher than that of individuals with a high school education level [7 (3–12)] and a primary education level [7 (3–12)] (Table 3).

Statistically significant differences were observed in the participants’ DASH and MEDAS scores according to income status. For DASH, the median value was higher in individuals whose income was greater than their expenses [38 (23–68)] than in individuals whose income was lower than their expenses [34 (23–59)] and in those whose income was equal to their expenses [34 (17–52)]. For MEDAS, the median value was higher in individuals whose income was lower than their expenses [8 (3–12)] than in those whose income was equal to their expenses [7 (3–11)] (Table 3).

Statistically significant differences were observed in the participants’ DASH and MEDAS scores according to smoking status. For DASH, the median value was higher in non-smokers [36 (17–68)] than in smokers [34 (21–50)]. For MEDAS, the median value was higher in non-smokers [8 (3–13)] than in smokers [7 (3–12)] and in those who had quit smoking [6 (3–11)] (Table 3).

Statistically significant differences were observed in MEDAS scores according to the participants’ alcohol consumption status. For MEDAS, the median value was higher in individuals who did not consume alcohol [8 (3–13)] than in those who had quit alcohol consumption [6 (3–11)] (Table 3).

Statistically significant differences were observed in MEDAS scores according to the participants’ meal-skipping status. For MEDAS, the median value was higher in individuals who skipped meals [8 (3–12)] than in individuals who did not skip meals [7 (3–13)] (Table 3).

Statistically significant differences were observed in the participants’ DASH and MEDAS scores according to daily water intake. For DASH, the median value was higher in individuals who consumed 2 L/day or more of water [37 (23–61)] than in those who consumed 1.5–2 L/day [36 (22–68)] and those who consumed 0–1.5 L/day [32 (17–56)]; the median value of individuals who consumed 1.5–2 L/day [36 (22–68)] was also higher than that of those who consumed 0–1.5 L/day [32 (17–56)]. For MEDAS, the median value was higher in individuals who consumed 2 L/day or more of water [8 (3–13)] than in those who consumed 1.5–2 L/day [7 (3–12)] and those who consumed 0–1.5 L/day [7 (3–12)] (Table 3).

A significant, negative, and weak relationship was observed between participants’ BMI and DASH scores, whereas a significant, negative, and very weak relationship was observed between BMI and MEDAS scores (*p* < 0.001; *p* < 0.01). As BMI increased, DASH and MEDAS scores tended to decrease (Table 3).

A statistically significant, negative, and weak relationship was observed between participants’ waist-to-hip ratio and DASH and MEDAS scores (*p* < 0.001). As this ratio increased, DASH and MEDAS scores tended to decrease (Table 3).

The results of the multiple linear regression analysis showed that all models were statistically significant (Model 1: F = 91.935, *p* < 0.001; Model 2: F = 85.356, *p* < 0.001; Model 3: F = 59.680, *p* < 0.001; Model 4: F = 69.086, *p* < 0.001). Model 1 explained 77.7% of the variance in the dependent variable (adjusted R^2^ = 0.777; R^2^ = 0.786), Model 2 explained 76.4% (adjusted R^2^ = 0.764; R^2^ = 0.773), Model 3 explained 69.3% (adjusted R^2^ = 0.693; R^2^ = 0.704), and Model 4 explained 72.3% (adjusted R^2^ = 0.723; R^2^ = 0.734). Whether there was a multicollinearity problem among the variables was examined using VIF and tolerance values. It was observed that the VIF values were below 5 and the tolerance values were above 0.20 for all variables, indicating that there was no multicollinearity problem in any of the models. When the residual analyses were examined, the standardized residual values were found to be within the acceptable range of ±3, and the Cook’s Distance values were below 1. These findings indicate that the model assumptions were met. In addition, the effects of the variables on the dependent variable were evaluated by including all variables in each model simultaneously (Table 4).

It was determined that the participants’ age, educational status, income status, smoking status, and alcohol consumption status had statistically significant effects on Oktem-VMPT total recall scores (*p* < 0.05; *p* < 0.01; *p* < 0.001). When the results were examined, it was observed that literate individuals had recall scores that were 4.505 units lower than those of individuals with a postgraduate education level, individuals with a primary education level had recall scores that were 4.980 units lower than those of individuals with a postgraduate education level, and individuals with a high school education level had recall scores that were 2.605 units lower than those of individuals with a postgraduate education level. In addition, individuals whose income was lower than their expenses had recall scores that were 0.766 units lower, and individuals whose income was equal to their expenses had recall scores that were 0.738 units lower than those of individuals whose income was greater than their expenses. It was also observed that non-smokers had recall scores that were 0.431 units higher than those of smokers, whereas individuals who had quit smoking had recall scores that were 1.147 units lower than those of smokers. Individuals who did not consume alcohol had recall scores that were 0.835 units higher, and individuals who had quit alcohol consumption had recall scores that were 1.954 units higher, than those of individuals who consumed alcohol (Table 4).

It was determined that the participants’ sex, age, educational status, and smoking status had statistically significant effects on TMT-A scores (*p* < 0.05; *p* < 0.01; *p* < 0.001). When the results were examined, it was found that men had TMT-A scores that were 1.369 units higher than those of women, a one-unit increase in age corresponded to a 0.313-unit increase in the TMT-A score, literate individuals had TMT-A scores that were 13.159 units higher than those of individuals with a postgraduate education level, individuals with a primary education level had TMT-A scores that were 9.727 units higher than those of individuals with a postgraduate education level, individuals with a high school education level had TMT-A scores that were 5.906 units higher than those of individuals with a postgraduate education level, and individuals with a university education level had TMT-A scores that were 1.335 units higher than those of individuals with a postgraduate education level. It was also determined that non-smokers had TMT-A scores that were 1.416 units lower than those of smokers (Table 4).

It was determined that the participants’ age, educational status, and smoking status had statistically significant effects on TMT-B scores (*p* < 0.05; *p* < 0.01; *p* < 0.001). When the results were examined, it was found that a one-unit increase in age corresponded to a 0.642-unit increase in the TMT-B score, literate individuals had TMT-B scores that were 27.595 units higher than those of individuals with a postgraduate education level, individuals with a primary education level had TMT-B scores that were 20.527 units higher than those of individuals with a postgraduate education level, and individuals with a high school education level had TMT-B scores that were 12.616 units higher than those of individuals with a postgraduate education level. It was also determined that non-smokers had TMT-B scores that were 3.185 units lower than those of smokers (Table 4).

It was determined that the participants’ age, educational status, and smoking status had statistically significant effects on the Total TMT score (*p* < 0.01; *p* < 0.001). When the results were examined, it was found that a one-unit increase in age corresponded to a 0.954-unit increase in the Total TMT score, literate individuals had Total TMT scores that were 40.754 units higher than those of individuals with a postgraduate education level, individuals with a primary education level had Total TMT scores that were 30.255 units higher than those of individuals with a postgraduate education level, and individuals with a high school education level had Total TMT scores that were 18.522 units higher than those of individuals with a postgraduate education level. It was also determined that non-smokers had Total TMT scores that were 4.601 units lower than those of smokers (Table 4).

A significant, negative, and weak relationship was found between men’s DASH scores and the Oktem-VMPT subcomponents “Learning Error Score” and “LTM Error Score” (*p* < 0.002). When the findings were evaluated, it was observed that increases in DASH scores were associated with a tendency toward decreases in the “Learning Error Score” and “LTM Error Score” of the Oktem-VMPT subcomponents (Table 5).

A significant, negative, and weak relationship was found between men’s MEDAS scores and the “Learning Error Score” subcomponent of the Oktem-VMPT (*p* < 0.002). When the findings were evaluated, it was observed that increases in MEDAS scores were associated with a tendency toward decreases in the “Learning Error Score” of the Oktem-VMPT subcomponents (Table 5).

A significant, positive, and weak relationship was found between women’s DASH scores and the Oktem-VMPT subcomponent scores for “Immediate Memory,” “Spontaneous Recall,” and “Recognition” (*p* < 0.002). A significant, negative, and weak relationship was found between women’s DASH scores and the Oktem-VMPT subcomponent scores for “Learning Error Score” and “LTM Error Score,” as well as the TMT subcomponent scores for TMT-A and TMT-B, and the Total TMT score (*p* < 0.002). When the findings were evaluated, it was observed that increases in DASH scores were associated with a tendency toward increases in the “Immediate Memory” and “Spontaneous Recall” scores, whereas the “Learning Error Score,” “LTM Error Score,” TMT-A, TMT-B, and Total TMT scores tended to decrease (Table 5).

A significant, positive, and weak relationship was found between participants’ DASH scores and the Oktem-VMPT subcomponent score for “Spontaneous Recall” (*p* < 0.002). Significant, negative, and weak relationships were found between DASH scores and the Oktem-VMPT subcomponent scores for “Learning Error Score” and “LTM Error Score,” as well as the TMT subcomponent scores for TMT-A and TMT-B, and the Total TMT score (*p* < 0.002). Significant, positive, but very weak relationships were also found between DASH scores and the Oktem-VMPT subcomponent score for “Immediate Memory” (*p* < 0.002). When the findings were evaluated, increases in DASH scores were observed to be associated with a tendency toward increases in the “Immediate Memory” and “Spontaneous Recall” scores, whereas the “Learning Error Score,” “LTM Error Score,” TMT-A, TMT-B, and Total TMT scores tended to decrease (Table 5).

Significant, positive, but very weak relationships were found between participants’ MEDAS scores and the Oktem-VMPT subcomponent scores for “Reaching Criterion,” “Highest Learning,” “Spontaneous Recall,” “Recognition,” and “Total Recall” (*p* < 0.002). In addition, a significant, negative, and likewise very weak correlation was identified between MEDAS scores and the Oktem-VMPT subcomponent score for “Learning Error Score” (*p* < 0.002). When the findings were evaluated, as MEDAS scores increased, the scores for “Reaching Criterion,” “Highest Learning,” “Spontaneous Recall,” “Recognition,” and “Total Recall” tended to increase, whereas the “Learning Error Score” tended to decrease (Table 5).

## 4. Discussion

In the study, it was determined that as dietary scores (DASH and MEDAS) increased, certain learning errors decreased in men; in other words, a significant relationship was found between diet quality and memory performance. It has been reported that increases in DASH and Mediterranean-style dietary scores reduce the rate of cognitive decline, thereby supporting the positive relationship between diet quality and cognitive performance [30]. Similarly, adherence to a Mediterranean-style diet has been reported to be associated with more favorable changes in cognitive functions [31]. In addition, systematic reviews have also supported that high adherence to the DASH, Mediterranean, and MIND diets is associated with better cognitive performance and slower cognitive decline [32]. It has also been reported that an increase in the MIND diet score is associated with slower cognitive decline and better performance across different cognitive domains [33]. Finally, high adherence to DASH and other healthy dietary models has also been reported to reduce the risk of subjective cognitive decline and improve objective cognitive performance [34]. This may be explained by the fact that the neuroprotective nutrients contained in the DASH and Mediterranean diets reduce oxidative stress and neuroinflammation, improve cerebral blood flow by supporting vascular health, and positively affect neurotransmitter balance through the gut–brain axis.

In the study, it was determined that as women’s DASH scores increased, the Oktem-VMPT subcomponent scores for “Immediate Memory” and “Spontaneous Recall” increased; in contrast, the scores for “Learning Error Score,” “LTM Error Score,” TMT-A, TMT-B, and Total TMT decreased. This finding is consistent with the study reporting that the Mediterranean, DASH, and MIND diets may have potential protective effects in reducing cognitive impairment and supports the relationship between healthy dietary patterns and better cognitive performance [35]. This may be explained by the fact that the DASH diet reduces oxidative stress and neuroinflammation through its neuroprotective nutrients, improves cerebral blood flow by supporting vascular health, and lowers the error rate in memory encoding by strengthening prefrontal cortex functions; these effects are thought to be observed more prominently in women together with the neuroprotective contribution of estrogen.

In the study, it was determined that as DASH scores increased, the Oktem-VMPT subcomponent scores for “Immediate Memory” and “Spontaneous Recall” increased; in contrast, the scores for “Learning Error Score,” “LTM Error Score,” TMT-A, TMT-B, and Total TMT decreased, indicating improved cognitive performance. This finding is consistent with the literature. Various review studies have reported that the DASH, Mediterranean, and MIND diets may have potential protective effects in preserving cognitive function and preventing cognitive decline [17,35,36]. This may be explained by the fact that the DASH diet reduces oxidative stress and neuroinflammation through its neuroprotective nutrients, improves cerebral blood flow by regulating blood pressure, strengthens executive control mechanisms by supporting prefrontal cortex functions, and positively affects neurotransmitter balance through the gut–brain axis.

In the study, it was determined that as MEDAS scores increased, the Oktem-VMPT subcomponent scores for “Reaching Criterion,” “Highest Learning,” “Spontaneous Recall,” “Recognition,” and “Total Recall” increased, whereas the “Learning Error Score” decreased. Adherence to the Mediterranean diet has been reported to be associated with verbal memory performance, and this finding is consistent with the increase in learning and recall performance observed in our study as MEDAS scores increased [37]. Similarly, it has been reported that the Mediterranean diet may preserve cognitive functions and reduce cognitive decline through its antioxidant and omega-3 content, and this finding supports our results indicating that memory and learning performance improved as MEDAS scores increased [38]. Polyphenols modulate the gut microbiota, which communicates bidirectionally with the brain via the vagus nerve and immune pathways, influencing neurotransmitter synthesis—including serotonin and dopamine—thereby affecting mood-dependent memory encoding [32]. The DASH diet’s blood pressure-lowering properties protect cerebral microvascular integrity, supporting adequate perfusion of the prefrontal cortex and hippocampus, which are critical for executive function and episodic memory respectively [30]. Dietary patterns high in antioxidants also preserve insulin sensitivity, which is important for hippocampal neurogenesis and neuroprotection, given that insulin resistance has been shown to impair memory consolidation [32]. It should be noted, however, that the observed correlations between dietary scores and cognitive performance, while statistically significant, were largely very weak in magnitude (Spearman’s r ≈ 0.13–0.28). In a sample of 600 participants, even small effect sizes reach statistical significance, and caution is warranted in interpreting these associations as clinically meaningful. The proportion of variance in cognitive outcomes explained by dietary adherence alone appears modest, with sociodemographic variables—particularly age, educational level, and smoking status—emerging as stronger predictors in multivariate models. These findings should therefore be regarded as preliminary and hypothesis-generating rather than as a basis for definitive public health recommendations.

The mean BMI and waist-to-hip ratio of the majority of the participants were within the normal range, and the mean BMI and waist-to-hip ratio of men were higher than those of women. Similarly, previous studies have reported that men had higher mean BMI and waist-to-hip ratio values than women [39,40,41]. This may be explained by sex-related dietary behaviors and hormonal differences, as well as by men’s relatively higher muscle mass and biological predisposition to abdominal adiposity.

The mean total MEDAS score of the majority of the participants was 7.43, and the mean total MEDAS score of women was higher than that of men, indicating acceptable adherence to the Mediterranean diet in both sexes. Similarly, previous studies reported that individuals’ mean MEDAS scores were at a moderate level [42,43]. It has been reported that women’s MEDAS scores are higher than those of men [44]. In contrast, men’s MEDAS scores have been reported to be higher [45]. The participants’ mean total DASH scores were at a moderate level of diet quality, and the mean DASH score of women was higher than that of men, indicating a moderate level of diet quality in both sexes. Similarly, previous studies reported that women’s adherence to the DASH diet was higher than that of men [46,47]. This may be explained by the fact that women show greater interest in nutrition and health issues than men and, within the framework of traditional gender roles, have more opportunities to acquire knowledge about food content by assuming responsibility for meal preparation.

Women had higher scores than men in the Oktem-VMPT subcomponents “Learning Score,” “Reaching Criterion,” “Highest Learning,” “Spontaneous Recall,” “Recognition,” and “Total Recall.” Only the “Learning Error Score” was higher in men than in women. It was observed that women’s learning and recall performance was higher than that of men. A meta-analysis reported that women performed better than men particularly in verbal memory, recall, and recognition tasks [48]. Women also show better performance than men, especially in verbal learning and recall [49]. Although one study reported that memory distortions were independent of sex, it also emphasized that false recognition rates varied according to emotional valence and that negatively valenced stimuli could increase susceptibility to memory distortions [50]. This may be associated with men’s higher learning error scores and sex differences related to erroneous memory encoding. This difference is thought to be related to women’s adoption of more effective memory strategies when encoding verbal material, owing to the more effective functioning of brain regions associated with language processing.

Female participants were found to have lower TMT-A and TMT-B subcomponent scores and lower Total TMT scores than men. Some studies have reported that the effect of sex on Trail Making Test performance is limited and have emphasized that performance is more strongly associated with age and educational level [51,52,53]. Similarly, one study reported that women performed better than men in verbal learning and memory tasks, whereas no significant sex difference was found in terms of overall cognitive performance [54]. Nevertheless, the literature indicates that the effects of sex on cognitive performance may vary across domains. In this context, sex is reported not to be a determining factor in general intelligence, although differences may be observed in certain cognitive domains; women may perform better in attention and inhibition tasks, whereas men may show better performance in tasks requiring spatial and executive functions [55,56]. This may suggest that, because the Trail Making Test simultaneously assesses visual scanning, psychomotor speed, and executive functions, it may reflect men’s cognitive advantage in such tasks, and that this difference may be explained by sex-related domain specificity in cognitive performance.

Female participants had higher median DASH and MEDAS scores than men. This finding is consistent with many studies in the literature. Various studies have reported that women’s adherence to the Mediterranean diet and healthy eating behaviors is higher than that of men [57,58,59]. Similarly, it has been reported that women’s adherence to the DASH diet is higher than that of men and that, although overall adherence declines over time, this sex difference persists [46,60]. However, a systematic review reported that sex differences in adherence to the Mediterranean diet are inconsistent; some studies found higher adherence among women, some among men, whereas others reported no significant difference between the sexes [61]. This may be explained by women’s higher levels of awareness regarding nutrition and greater health-related motivation than men, their greater opportunity to acquire knowledge about food content by assuming responsibility for meal preparation within the framework of traditional gender roles, and the tendency of social pressures to direct women toward healthier food choices.

Participants aged 18–35 years had the highest median DASH and MEDAS scores. A statistically significant, negative, and very weak relationship was found between age and DASH scores, and as age increased, DASH scores were found to decrease. This finding is consistent with a study reporting that diet quality is influenced by age and life transitions and that significant changes in dietary behaviors may be observed during young adulthood [62]. However, the literature also includes findings that contradict this result. Specifically, some studies have reported that adherence to the DASH diet and the Mediterranean diet increases with age [46,58,63]. On the other hand, there are also studies reporting that adherence to the Mediterranean diet may decrease over time in older age groups [64]. This may be explained by the possibility that, as age increases, time constraints associated with work and family life and rising stress levels may negatively affect healthy eating behaviors; meanwhile, the contradictory findings in the literature may be explained by differences in sample characteristics and cultural dietary habits.

The median values for MEDAS and DASH were higher in single individuals than in married individuals. The literature reports that adherence to the Mediterranean diet is influenced by sociodemographic factors such as marital status, and that relationship status and lifestyle in particular may shape dietary behaviors [65,66]. However, one study reported that marital status affects eating behaviors and that more irregular eating behaviors may be observed among single individuals, which partially contradicts our findings [67]. On the other hand, it has also been reported that household structure affects diet quality and health indicators, and that dietary behaviors differ in single-person households [68]. This may suggest that time constraints arising from work and family responsibilities among married individuals may have negatively affected healthy eating behaviors.

Postgraduate-level individuals had the highest median MEDAS and DASH scores. This finding is consistent with the literature. Various studies have reported that individuals with higher levels of education show greater adherence to both the Mediterranean diet and the DASH diet [42,69,70]. Similarly, higher educational attainment has also been reported to be positively associated with knowledge of the DASH diet and the level of adherence to it [71]. This may be explained by the fact that the socioeconomic advantages associated with the high level of education attained through postgraduate education facilitate access to healthy foods and increase motivation toward long-term health goals.

In the present study, adherence to the DASH diet was found to be highest among individuals whose income was greater than their expenses, and this finding is consistent with the literature. Similarly, it has been reported that DASH scores are higher in high-income individuals and that there is a significant relationship between income level and adherence to the DASH diet [72]. Likewise, it has also been reported that individuals with a higher socioeconomic status show greater adherence to the DASH diet and that income level is positively associated with diet quality [73]. However, the finding that the MEDAS score was highest among individuals whose income was lower than their expenses contradicts a study reporting that individuals with higher income levels have greater adherence to the Mediterranean diet [74]. The higher adherence to the DASH diet among individuals whose income was greater than their expenses may be explained by the fact that the structured nature of this diet, based on specific food groups, may be more easily sustained through conscious food choices by individuals who are more economically advantaged. In contrast, the finding that the MEDAS score was higher among individuals whose income was lower than their expenses may be explained by the fact that low-income individuals may have adopted a more traditional and home-oriented dietary pattern by limiting eating out and the consumption of convenience foods, as well as by the confounding effect of demographic variables.

In this study, the median DASH and MEDAS values were found to be highest among non-smokers. This finding is consistent with the literature. Various studies have reported that adherence to the DASH and MEDAS diets is higher among non-smokers, that smoking is negatively associated with diet quality, and that not smoking is positively associated with MEDAS [46,72,75]. Similarly, it has also been reported that higher diet quality is observed in non-smokers and that smoking may reduce the protective effect of diet [76]. The finding that the median MEDAS value was higher among individuals who did not consume alcohol is also consistent with the literature. Alcohol consumption has been reported to be associated with non-adherence to the Mediterranean diet and lower MEDAS adherence; it has also been reported that not smoking is positively associated with MEDAS, whereas alcohol consumption is associated with lower diet adherence [46,77]. The finding that the median MEDAS value was higher among individuals who did not skip meals is consistent with a study reporting that MEDAS scores are significantly associated with the number of snacks consumed [78]. However, there are also studies reporting that MEDAS scores do not show a significant relationship with meal skipping [79]. These findings may be explained by the tendency of not smoking, not consuming alcohol, and eating regular meals to cluster together with positive health behaviors such as healthy eating and high health awareness. In addition, smoking may impair taste and smell perception, while alcohol may increase appetite, which in turn promotes a tendency toward high-calorie and processed foods. Meal irregularity may also make it more difficult to adequately include the core components of the Mediterranean diet, all of which are factors that negatively affect diet quality.

The median DASH and MEDAS values were highest among individuals who consumed 2 L/day or more of water. One study reported that water consumption is positively associated with higher diet quality [80]. This may be explained by the fact that water consumption reflects general health awareness and a healthy lifestyle.

As BMI and waist-to-hip ratio increased, decreases in DASH and MEDAS scores were observed. This finding is consistent with the literature. It has been reported that as adiposity indicators such as BMI and waist-to-hip ratio increase, diet quality scores such as DASH and MEDAS decrease [81]. Similarly, many studies have reported that as adherence to the Mediterranean diet increases, adiposity indicators such as BMI, waist circumference, and waist-to-hip ratio decrease, and that higher diet quality is associated with lower anthropometric values [82,83,84,85]. In addition, it has been reported that as adherence to the Mediterranean diet increases, the risk of abdominal obesity decreases, whereas abdominal obesity is more prevalent in individuals with lower diet adherence; overall, higher diet quality is emphasized to be associated with lower abdominal adiposity [86,87]. Furthermore, it has also been reported that as adherence to the DASH diet increases, waist-to-hip ratio decreases [88]. This may be explained by the fact that the high fiber and anti-inflammatory nutrient content of the DASH and Mediterranean diets helps reduce abdominal adiposity by balancing energy intake, whereas individuals with higher adiposity may tend to move away from these dietary patterns due to their greater inclination toward processed foods and saturated fat consumption.

It was determined that as participants’ educational level increased, Oktem-VMPT total recall scores tended to increase, with the highest mean score observed in individuals with a postgraduate education level and the lowest mean score observed in individuals with a primary education level. This finding is consistent with the literature. It has been reported that as educational level increases, memory performance improves and brain volume is higher [89]. Similarly, it has been reported that individuals who receive university education show improved cognitive test performance, particularly in the domains of verbal learning, memory, and episodic memory, and that even education received at an older age may preserve or improve cognitive functions [90]. It has also been reported that educational level enhances memory performance, although it does not affect all cognitive domains to the same extent [91]. In addition, it is emphasized that a higher educational level reduces the risk of cognitive impairment and is the strongest determinant of cognitive health [92]. It is thought that the cognitive reserve mechanism underlies this relationship, and it has been reported that as hippocampal volume increases, delayed recall performance improves, that this relationship is stronger in individuals with a higher educational level, and therefore that a higher educational level supports more effective use of the existing brain structure [93]. This may be explained by the fact that a higher educational level increases cognitive reserve and supports more effective use of the existing brain structure, that advanced learning strategies acquired during the educational process strengthen memory encoding and recall processes, and that lifelong cognitive activity supports neuroplasticity and positively affects memory functions.

It was determined that individuals whose income was greater than their expenses had higher recall scores. This finding is consistent with the literature. It has been reported that low socioeconomic status is associated with poorer memory performance and more rapid cognitive decline, whereas high socioeconomic status is associated with better memory performance [94,95]. Similarly, it has been reported that socioeconomic disadvantage is associated with poorer performance across all cognitive domains, including memory, and that lifelong low socioeconomic status is linked to poorer cognitive performance [96]. In addition, it has been reported that as financial status worsens, memory performance decreases and cognitive decline accelerates [97]. It is also emphasized that environmental variables such as economic status and education play a determining role in working memory [98]. This may be explained by the fact that economic hardship may negatively affect hippocampal structure through chronic stress, that individuals with low income may have limited access to quality nutrition and healthcare services, and that higher income may strengthen cognitive reserve by increasing opportunities for cognitive stimulation.

Smoking and alcohol use were found to be associated with lower recall/memory performance. It has been reported that individuals who smoke show lower levels of verbal learning and recall, slower attention and processing speed, and greater cognitive impairment as the amount and duration of smoking increase [99]. Similarly, it has been reported that individuals with a history of alcohol dependence have lower attention and memory performance, markedly weakened learning and recall abilities, and significantly greater cognitive impairment compared with healthy controls [100]. In addition, it has been reported that the combined use of smoking and alcohol exerts a more pronounced negative effect on cognitive performance, with the lowest cognitive performance observed in the group using both substances and the highest performance observed in the group using neither substance [101]. This may be explained by the fact that the neurotoxic effects of smoking and alcohol adversely affect the hippocampal structure and the cholinergic system, reduce cerebral blood flow and thereby decrease the amount of oxygen reaching the brain, and disrupt neurotransmitter balance, thereby weakening memory encoding and recall processes; these negative effects are thought to become cumulative when both substances are used together.

Male participants had higher mean TMT-A scores than women. It has been reported that TMT performance is affected by the sex variable and that sex may influence cognitive performance [102]. Similarly, it has been reported that men perform better than women in certain test sections requiring psychomotor speed and attention, and that they are particularly more successful in sustaining attention [103]. In addition, it has been reported that male students obtained higher scores than female students in executive functions, and that this difference was statistically significant both in total executive function scores and in subdimensions [104]. On the other hand, the literature also includes findings that contradict this result. It has been reported that, in the TMT, no significant sex difference was found in Part A, whereas a significant difference was identified in Part B [105]. There are also studies reporting that the sex variable does not have a significant effect on scores in the TMT [106]. In another study, it was reported that women performed better on TMT-A, whereas men performed better on TMT-B, and that attention performance may be affected differently depending on sex [107]. This may be explained by the possibility that TMT-A, due to its structure requiring visual scanning speed and psychomotor speed, may reflect men’s cognitive advantage in such tasks, and that this difference may be partially explained by the positive effects of testosterone on psychomotor speed and sustained attention.

As participants’ age increased, TMT-A, TMT-B, and Total TMT scores also increased. This finding is consistent with a study reporting that performance decreases linearly across all measurements as age increases [108]. However, the literature also includes studies reporting that TMT performance is not significantly affected by age [106]. This may be explained by the decline in processing speed and attentional capacity due to structural changes occurring in the prefrontal cortex and related brain regions during the normal aging process.

As participants’ educational level increased, TMT-A, TMT-B, and Total TMT scores decreased. This finding is consistent with studies reporting that TMT performance decreases with lower educational level and that higher educational level is associated with better TMT performance [108,109]. However, the literature also includes studies reporting that no direct relationship exists between educational level and cognitive test performance [110]. This may be explained by the fact that higher educational level increases cognitive reserve, thereby supporting the cognitive processes required by the TMT, such as attention, visual scanning, and psychomotor speed, and that symbol recognition and rapid information-processing skills acquired during the educational process may positively affect test performance.

In the study, it was determined that non-smoking participants had lower TMT-A, TMT-B, and Total TMT scores than smoking participants, indicating better performance. This finding is consistent with the literature. It has been reported that individuals who smoke exhibit neurocognitive impairments in attention, executive functions, and verbal memory, and that these impairments are related to the duration and dose of smoking [99]. Similarly, meta-analytic evidence also supports that chronic smoking is associated with impairment across many neuropsychological domains, including attention, cognitive flexibility, and memory [111]. In addition, chronic smoking has been reported to lead to impairment in general cognitive functions and abstract reasoning performance [112]. Lower performance has also been reported in smokers on tasks sensitive to inhibitory control and orbitofrontal cortex functions [113]. However, the literature also includes studies reporting that smoking negatively affects TMT-A performance, whereas its effect on TMT-B performance remains limited [114,115]. This may be explained by the fact that chronic smoking reduces cerebral blood flow and adversely affects dopaminergic and cholinergic systems, and that these neurotoxic effects increase in proportion to the duration and dose of use, thereby weakening attentional capacity and psychomotor speed.

The differential effects observed across cognitive domains—specifically, the stronger associations with verbal memory (Oktem-VMPT) compared to executive function and visual scanning speed (TMT)—warrant specific mechanistic consideration. Verbal memory and learning, as measured by the Oktem-VMPT, depend heavily on hippocampal integrity and cholinergic neurotransmission, both of which are sensitive to anti-inflammatory dietary components and omega-3-mediated synaptic support. In contrast, TMT performance relies more on white matter connectivity, dopaminergic signaling, and prefrontal network integrity, which may require longer-term or higher-intensity dietary exposure to show measurable change—particularly in younger, healthier populations such as ours. This may partly explain why diet–TMT associations did not reach significance in our regression models after covariate adjustment.

In this context, the findings of Koutsonida et al. (2021), who reported a significant association between Mediterranean diet adherence and TMT performance in a Greek cohort, are relevant [116]. While their findings broadly support a diet–executive function link, notable differences between their study and ours may explain the discrepancy. Their sample consisted of older adults, in whom vascular and neuroinflammatory pathways may already have produced detectable cognitive differences amenable to dietary modulation, whereas our sample’s younger mean age (36 years) and generally intact TMT performance may have limited the range of detectable diet-related variation. Furthermore, the stronger predictive effects of age and educational level on TMT performance in our sample may have attenuated the independent contribution of diet in multivariate models. Future longitudinal studies in aging cohorts would be better positioned to detect diet–executive function associations over time.

At the molecular level, emerging evidence suggests that dietary bioactive compounds, including polyphenols and omega-3 fatty acids found abundantly in both the Mediterranean and DASH dietary patterns, may influence RNA splicing regulatory mechanisms involved in neurodegeneration [117]. Alternative splicing dysregulation has been implicated in the pathogenesis of several neurodegenerative conditions, and dietary modulation of these pathways may represent an underexplored mechanism through which healthy dietary patterns exert neuroprotective effects beyond classical anti-inflammatory and vascular pathways. While our study was not designed to assess molecular mechanisms, these considerations provide important context for the behavioral associations we observed and highlight promising directions for future mechanistic research.

The stronger diet–cognition associations observed in women in our study—with DASH scores significantly predicting immediate memory, spontaneous recall, and TMT performance in women but not men after Bonferroni correction—may reflect the modulatory role of estrogen. Estrogen enhances the neuroprotective effects of dietary antioxidants through upregulation of brain-derived neurotrophic factor (BDNF) expression and supports cholinergic functioning, potentially amplifying the cognitive benefits of healthy dietary patterns in women [35].

A note on the cognitive tools employed is warranted. The Oktem-VMPT is a validated instrument for multidimensional assessment of verbal learning and memory in Turkish populations, and the TMT is a widely used measure of processing speed and executive function. However, both tools are performance-based measures sensitive to test-day variability, education-related familiarity effects, and motivational factors. The absence of neuroimaging data means that mechanistic interpretations remain inferential. Future studies incorporating structural MRI, functional connectivity measures, or biomarkers of neuroinflammation would substantially strengthen causal claims regarding dietary effects on specific cognitive domains. In this regard, recent advances in multimodal radiomics have demonstrated that integrating complementary imaging modalities can substantially enhance predictive accuracy for complex clinical outcomes; adapting such analytical frameworks to diet–cognition research may offer novel mechanistic insights [118].

## 5. Strengths and Limitations

Strengths of the Study: This study fills an important gap in the literature by jointly examining the effects of adherence to the DASH diet and the Mediterranean diet on cognitive performance. The limited use of these dietary models together with the Oktem-VMPT and TMT increases the originality of the study. The large sample size and the use of valid measurement tools support the validity of the findings, and the results obtained make a meaningful contribution to national preventive public health policies related to neurological and psychological health.

In the literature, various dietary patterns have been studied separately. While this approach introduces some originality by combining these dietary patterns, it also reveals the diverse dietary habits found across different regions of Turkey. For instance, the eastern regions primarily consume a diet rich in red meat and animal-derived butter, while the Central Anatolia region focuses on carbohydrate-rich baked goods. This study addresses a knowledge gap by increasing awareness about the effects of the DASH and Mediterranean diets on cognitive performance.

Limitations of the Study: The cross-sectional design of this study limits causal inferences. Self-report-based data increase the risk of recall bias and other forms of bias. The inability to fully control potential confounding variables and the short-term assessment of dietary adherence are among the other limitations that restrict the interpretation of the findings. In addition, the DASH-Q evaluates dietary intake over the previous 7 days; therefore, the observed associations may reflect recent dietary behavior rather than long-term habitual dietary patterns that are more likely to influence cognitive performance. Furthermore, although DASH and MEDAS scores were compared across age subgroups (18–35, 36–50, and 51–65 years), age-stratified analyses of the diet–cognition relationship were not conducted, as dividing the sample into subgroups would reduce statistical power. This remains a limitation of the present study, and future research with larger samples is encouraged to explore whether the associations between dietary adherence and cognitive performance differ across age groups.

Furthermore, this temporal limitation may disproportionately affect younger adults in the sample, whose week-to-week dietary patterns tend to be more variable compared to older age groups. This variability may introduce additional measurement error in younger participants’ DASH scores, potentially confounding age-related comparisons of diet quality and cognitive performance.

## 6. Conclusions

This study comprehensively examined the relationship between diet quality and cognitive performance in young and middle-aged adults. The findings reveal that higher adherence to the DASH and Mediterranean diets was associated with better verbal memory and learning performance in bivariate analyses; however, these associations, along with those with executive function and visual scanning speed measured by the TMT, were attenuated after adjustment for sociodemographic covariates in multivariate models. These results suggest that diet’s contribution to cognitive health may be domain-specific and partly mediated through sociodemographic pathways. Women demonstrated higher diet quality and verbal memory performance compared to men, while men showed relatively better performance on tasks requiring psychomotor speed and executive functions. Sociodemographic and lifestyle factors—including educational level, income, smoking, and alcohol use—were found to significantly influence both diet quality and cognitive functioning. Higher education and income were associated with better dietary behaviors and cognitive performance, whereas smoking and alcohol consumption were linked to adverse outcomes in both domains. Healthy lifestyle behaviors such as adequate water intake, regular meal consumption, and non-smoking were found to cluster together with higher diet quality and to support cognitive health. The inverse association between increasing BMI and waist-to-hip ratio and diet quality scores likely reflects a reciprocal interaction between anthropometric indicators and dietary behaviors, though the cross-sectional design precludes causal interpretation.

In conclusion, improving adherence to evidence-based dietary models such as the DASH and Mediterranean diets holds significant potential for preserving cognitive health, particularly with regard to verbal memory and learning. The neuroprotective mechanisms underlying these associations—including reduction of neuroinflammation, modulation of gut microbiota–brain axis signaling, and support of cerebrovascular integrity—warrant further investigation at the molecular level. Given that gender, age, education, and lifestyle factors play determining roles in this relationship, public health interventions should be designed with a multidimensional approach that accounts for individual differences. Future studies employing longitudinal designs, larger and more diverse samples, and neuroimaging or biomarker-based methods are recommended to clarify the mechanistic and domain-specific dimensions of diet–cognition associations.

## Figures and Tables

**Table 1:** Descriptive statistics of demographic, health, lifestyle, and dietary habit findings of the participants by sex.

	Male (*n* = 257)		Female (*n* = 343)		Total (*n* = 600)	
**Marital Status**	***n***	**%**	***n***	**%**	***n***	**%**
Married	174	67.7	165	48.1	339	56.5
Single	83	32.3	178	51.9	261	43.5
**Educational Status**						
Literate	24	9.3	40	11.7	64	10.7
Primary Education	25	9.7	21	6.1	46	7.7
High School	48	18.7	51	14.9	99	16.5
University	129	50.2	185	53.9	314	52.3
Postgraduate	31	12.1	46	13.4	77	12.8
**Income Status**						
Income lower than expenses	41	16.0	162	47.2	203	33.8
Income equal to expenses	79	30.7	66	19.2	145	24.2
Income greater than expenses	137	53.3	115	33.5	252	42.0
**Smoking Status**						
Yes	88	34.2	40	11.7	128	21.3
No	154	59.9	299	87.2	453	75.5
Quit	15	5.8	4	1.2	19	3.2
**Alcohol Consumption Status**						
Yes	15	5.8	10	2.9	25	4.2
No	229	89.1	333	97.1	562	93.7
Quit	13	5.1	0	0.0	13	2.2
**Chronic Disease Status**						
Yes	31	12.1	50	14.6	81	13.5
No	226	87.9	293	85.4	519	86.5
**Regular Medication Use**						
Yes	9	3.5	17	5.0	26	4.3
No	248	96.5	326	95.0	574	95.7
**Regular Dietary Supplement Use**						
Yes	29	11.3	47	13.7	76	12.7
No	228	88.7	296	86.3	524	87.3
**Meal Skipping Status**						
Yes	150	58.4	259	75.5	409	68.2
No	107	41.6	84	24.5	191	31.8
**Daily Water Intake**						
0 ≤ 1.5 L/day	49	19.1	51	14.9	100	16.7
1.5 ≤ 2 L/day	125	48.6	155	45.2	280	46.7
≥2 L/day	83	32.3	137	39.9	220	36.7

**Table 2:** Descriptive statistics of age, anthropometric measurement values, DASH and MEDAS scores, and Oktem-VMPT and TMT measurements of the participants by sex.

	Male		Female		Total			
	$\bar{X}$ ± *SS*	Median (Min–Max)	$\bar{X}$ ± *SS*	Median (Min–max)	$\bar{X}$ ± *SS*	Median (Min–Max)	U	*p*
**Age**	39.09 ± 13.03	38 (18–65)	33.75 ± 12.36	29 (18–64)	36.04 ± 12.91	32 (18–65)	**33,411.5**	**<0.001** ***
**BMI**	26.24 ± 3.04	25.9 (20–42.8)	24.69 ± 4.26	22.8 (17.3–37.7)	25.35 ± 3.86	24.7 (17.3–42.8)	**28,621.5**	**<0.001** ***
**Waist-to-Hip Ratio**	0.86 ± 0.05	0.9 (0.8–1)	0.79 ± 0.07	0.8 (0.7–1)	0.82 ± 0.07	0.8 (0.7–1)	**15,358.5**	**<0.001** ***
**Total MEDAS**	6.49 ± 1.80	6 (3–12)	8.13 ± 1.59	8 (4–13)	7.43 ± 1.87	8 (3–13)	**21,961**	**<0.001** ***
**Total DASH**	35.44 ± 5.95	35 (21–54)	36.90 ± 7.50	36 (17–68)	36.27 ± 6.91	36 (17–68)	**39,467**	**0.028** *
**Oktem-VMPT subcomponents**								
**Immediate Memory**	5.46 ± 1.16	6 (3–9)	5.65 ± 1.24	6 (2–9)	5.57 ± 1.21	6 (2–9)	**39,383**	**0.020** *
**Learning Score**	104.57 ± 19.15	109 (6–141)	109.76 ± 17.48	117 (55–142)	107.54 ± 18.38	112 (6–142)	**36,934.5**	**<0.001** ***
**Reaching Criterion**	13.28 ± 2.06	14 (8–15)	13.88 ± 1.88	15 (7–15)	13.62 ± 1.98	15 (7–15)	**35,762**	**<0.001** ***
**Highest Learning**	13.28 ± 2.06	14 (8–15)	13.88 ± 1.88	15 (7–15)	13.62 ± 1.98	15 (7–15)	**35,762**	**<0.001** ***
**Learning Error Score**	0.93 ± 0.80	1 (0–3)	0.80 ± 0.83	1 (0–4)	0.86 ± 0.82	1 (0–4)	**39,639.5**	**0.023** *
**Spontaneous Recall**	11.81 ± 2.18	13 (6–15)	12.38 ± 2.09	13 (5–15)	12.14 ± 2.15	13 (5–15)	**36,371**	**<0.001** ***
**Recognition**	13.35 ± 2.08	14 (8–15)	13.94 ± 1.81	15 (7–15)	13.69 ± 1.95	15 (7–15)	**36,845.5**	**<0.001** ***
**Total Recall**	25.16 ± 4.18	27 (14–30)	26.32 ± 3.79	28 (12–30)	25.82 ± 4.00	28 (12–30)	**36,015.5**	**<0.001** ***
**LTM Error Score**	0.26 ± 0.50	0 (0–2)	0.23 ± 0.48	0 (0–2)	0.24 ± 0.49	0 (0–2)	42,847.5	0.412
**TMT-A**	33.34 ± 8.96	31 (20–64)	31.75 ± 9.55	28 (20–68)	32.43 ± 9.32	29 (20–68)	**37,959.5**	**0.004** **
**TMT-B**	72.02 ± 20.02	67 (45–138)	68.01 ± 20.26	61 (42–142)	69.73 ± 20.24	63 (42–142)	**37,828**	**0.003** **
**Total TMT**	105.35 ± 28.80	99 (65–202)	99.76 ± 29.63	89 (62–210)	102.16 ± 29.38	93 (62–210)	**37,831**	**0.003** **

TMT-A: Processing Speed Based on Visual Scanning Ability; TMT-B: Ability to Shift Set Between Stimulus Sets and Track Sequencing; LTM: Long-Term Memory; U: Mann–Whitney U Test Statistically significant differences were observed in the participants’ DASH and MEDAS scores according to sex. For DASH, the median value was higher in women [36 (17–68)] than in men [35 (21–54)], and similarly, for MEDAS, the median value was also higher in women [8 (4–13)] than in men [6 (3–12)] (Table 3). * *p* < 0.05, ** *p* < 0.01, *** *p* < 0.001.

**Table 3:** Comparison of DASH and MEDAS scores according to the participants’ demographic, health, lifestyle, dietary habit, and anthropometric characteristics.

		DASH Total	MEDAS Total
Male		35 (21–54)	6 (3–12)
Female		36 (17–68)	8 (4–13)
U		**U = 39,467**	**U = 21,961**
***p***		**0.028** *	**<0.001** ***
**Age Group**			
18–35 years		37 b (17–68)	8 b (3–13)
36–50 years		36 b (23–54)	7 a (4–11)
51–65 years		33.5 a (23–47)	7 a (3–12)
**H**		**H = 20.546**	**H = 11.058**
***p***		**<0.001** ***	**0.004** **
**Age**	**s**	**−0.111**	−0.078
	***p***	**0.007** **	0.058
**Marital Status**			
Married		35 (21–68)	7 (3–13)
Single		37 (17–61)	8 (3–12)
**U**		**U = 39,061.5**	**U = 37,289**
***p***		**0.014** *	**<0.001** ***
**Educational Status**			
Literate		32 a (24–53)	8 bc (4–12)
Primary Education		35 ab (23–47)	7 a (3–10)
High School		35 b (24–50)	7 a (3–12)
University		37 c (21–68)	7 b (3–12)
Postgraduate		38 c (17–59)	8 c (5–13)
**H**		**H = 31.741**	**H = 27.414**
***p***		**<0.001** ***	**<0.001** ***
**Income Status**			
Income lower than expenses		34 a (23–59)	8 b (3–12)
Income equal to expenses		34 a (17–52)	7 a (3–11)
Income greater than expenses		38 b (23–68)	8 ab (3–13)
**H**		**H = 35.427**	**H = 6.926**
***p***		**<0.001** ***	**0.031** *
**Smoking Status**			
Yes		34 a (21–50)	7 a (3–12)
No		36 b (17–68)	8 b (3–13)
Quit		36 ab (29–45)	6 a (3–11)
**H**		**H = 9.591**	**H = 23.889**
***p***		**0.008** **	**<0.001** ***
**Alcohol Consumption Status**			
Yes		35 (17–50)	7 ab (3–10)
No		36 (21–68)	8 b (3–13)
Quit		34 (29–40)	6 a (3–11)
H		H = 1.861	H = 9.146
*p*		0.394	0.010 *
**Chronic Disease Status**			
Yes		36 (17–59)	7 (4–11)
No		36 (21–68)	8 (3–13)
U		U = 20,606	U = 19,332
*p*		0.775	0.239
**Regular Medication Use**			
Yes		36 (24–45)	7 (4–10)
No		36 (17–68)	8 (3–13)
U		U = 7449.5	U = 6766.5
*p*		0.988	0.415
**Regular Dietary Supplement Use**			
Yes		35 (23–56)	7 (4–12)
No		36 (17–68)	8 (3–13)
U		U = 19,065	U = 19,240.5
*p*		0.548	0.630
**Meal Skipping Status**			
Yes		36 (17–68)	8 (3–12)
No		36 (21–56)	7 (3–13)
U		U = 37,910	U = 32,174.5
*p*		0.561	<0.001 ***
**Daily Water Intake**			
0 ≤ 1.5 L/day		32 a (17–56)	7 a (3–12)
1.5 ≤ 2 L/day		36 b (22–68)	7 a (3–12)
≥2 L/day		37 c (23–61)	8 b (3–13)
**H**		**H = 33.381**	**H = 23.790**
***p***		**<0.001** ***	**<0.001** ***
**BMI**	**s**	**−0.277**	**−0.129**
	***p***	**<0.001** ***	**0.002** **
**Waist-to-Hip Ratio**	**s**	**−0.332**	**−0.205**
	***p***	**<0.001** ***	**<0.001** ***

U: Mann–Whitney U Test; H: Kruskal–Wallis H Test; s: Spearman Rank Difference Correlation Coefficients * *p* < 0.05; ** *p* < 0.01; *** *p* < 0.001 a, b, c: The difference between medians that do not share a common letter is significant (*p* < 0.05).

**Table 4:** Analysis of the effects of participants’ demographic, lifestyle, dietary habits, anthropometric, DASH, and MEDAS scores on Oktem-VMPT and TMT performance measures.

Model			Unstandardized Coefficients		t	*p*	95% Confidence Interval for B		Multicollinearity Statistics	
			B	SE			Lower Bound	Upper Bound	Tolerance	VIF
**Oktem-VMPT**	(Constant)		29.638	1.688	**17.555**	<0.001 ***	26.322	32.954		
	**Sex (Ref: Female)**									
	Male		0.183	0.229	0.797	0.426	−0.267	0.633	0.462	2.166
	**Age**		**−0.163**	0.011	**−14.549**	<0.001 ***	−0.185	−0.141	0.283	3.528
	**Marital Status (Ref: Married)**									
	Single		−0.031	0.235	−0.132	0.895	−0.492	0.430	0.439	2.278
	**Educational Status (Ref: Postgraduate)**									
	Literate		**−4.505**	0.414	**−10.882**	<0.001 ***	−5.318	−3.692	0.364	2.750
	Primary Education		**−4.980**	0.454	**−10.969**	<0.001 ***	−5.872	−4.088	0.407	2.458
	High School		**−2.605**	0.329	**−7.925**	<0.001 ***	−3.250	−1.959	0.399	2.506
	University		−0.369	0.244	−1.514	0.131	−0.848	0.110	0.400	2.499
	**Income Status (Ref: Income Greater Than Expenses)**									
	Income Lower than Expenses		**−0.766**	0.231	**−3.308**	0.001 **	−1.220	−0.311	0.495	2.020
	Income Equal to Expenses		**−0.738**	0.224	**−3.292**	0.001 **	−1.178	−0.297	0.645	1.549
	**Smoking Status (Ref: Yes)**									
	No		**0.431**	0.215	**2.002**	0.046 *	0.008	0.855	0.692	1.446
	Quit		**−1.147**	0.565	**−2.032**	0.043 *	−2.256	−0.038	0.607	1.646
	**Alcohol Consumption Status (Ref: Yes)**									
	No		**0.835**	0.412	**2.027**	0.043 *	0.026	1.644	0.590	1.695
	Quit		**1.954**	0.764	**2.557**	0.011 *	0.453	3.455	0.480	2.084
	**Meal Skipping Status (Ref: Yes)**									
	No		0.249	0.171	1.455	0.146	−0.087	0.585	0.933	1.072
	**Daily Water Intake (Ref: ≥2 L/day)**									
	0 ≤ 1.5 L/day		−0.033	0.244	−0.133	0.894	−0.511	0.446	0.720	1.390
	1.5 ≤ 2 L/day		0.029	0.181	0.161	0.872	−0.326	0.384	0.731	1.368
	**BMI**		0.041	0.041	0.988	0.324	−0.040	0.122	0.231	4.322
	**Waist-to-Hip Ratio**		1.903	2.365	0.805	0.421	−2.742	6.548	0.224	4.464
	**Total DASH**		−0.015	0.013	−1.196	0.232	−0.041	0.010	0.754	1.327
	**Total MEDAS**		0.081	0.050	1.607	0.109	−0.018	0.179	0.677	1.476
	**TMT-A**	(Constant)	5.872	4.051	1.450	0.148	−2.084	13.829		
		**Sex (Ref: Female)**								
		Male	**1.369**	0.550	**2.489**	0.013 *	0.289	2.449	0.462	2.166
		**Age**	**0.313**	0.027	**11.615**	<0.001 ***	0.260	0.366	0.283	3.528
	**Marital Status (Ref: Married)**									
	Single		−0.022	0.563	−0.039	0.969	−1.128	1.084	0.439	2.278
	**Educational Status (Ref: Postgraduate)**									
	Literate		**13.159**	0.993	**13.248**	<0.001 ***	11.208	15.110	0.364	2.750
	Primary Education		**9.727**	1.089	**8.929**	<0.001 ***	7.588	11.867	0.407	2.458
	High School		**5.906**	0.789	**7.489**	<0.001 ***	4.357	7.454	0.399	2.506
	University		**1.335**	0.585	**2.282**	0.023 *	0.186	2.485	0.400	2.499
	**Income Status (Ref: Income Greater Than Expenses)**									
	Income Lower Than Expenses		0.942	0.555	1.697	0.090	−0.149	2.033	0.495	2.020
	Income Equal to Expenses		0.083	0.538	0.154	0.877	−0.973	1.139	0.645	1.549
	**Smoking Status (Ref: Yes)**									
	No		**−1.416**	0.517	**−2.739**	0.006 **	−2.431	−0.400	0.692	1.446
	Quit		0.251	1.355	0.186	0.853	−2.409	2.912	0.607	1.646
	**Alcohol Consumption Status (Ref: Yes)**									
	No		−1.771	0.988	−1.792	0.074	−3.713	0.170	0.590	1.695
	Quit		−2.153	1.833	−1.174	0.241	−5.754	1.448	0.480	2.084
	**Meal Skipping Status (Ref: Yes)**									
	No		−0.073	0.411	−0.177	0.860	−0.880	0.734	0.933	1.072
	**Daily Water Intake (Ref: ≥2 L/day and above)**									
	0 ≤ 1.5 L/day		0.452	0.585	0.773	0.440	−0.697	1.601	0.720	1.390
	1.5 ≤ 2 L/day		0.511	0.434	1.178	0.239	−0.341	1.362	0.731	1.368
	**BMI**		0.142	0.100	1.431	0.153	−0.053	0.338	0.231	4.322
	**Waist-to-Hip Ratio**		11.054	5.674	1.948	0.052	−0.091	22.198	0.224	4.464
	**Total DASH**		0.002	0.031	0.080	0.936	−0.058	0.063	0.754	1.327
	**Total MEDAS**		0.008	0.120	0.070	0.945	−0.228	0.245	0.677	1.476
	**TMT-B**	(Constant)	**33.655**	10.036	**3.353**	0.001 **	13.942	53.367		
		**Sex (Ref: Female)**								
		Male	1.138	1.362	0.835	0.404	−1.538	3.814	0.462	2.166
		**Age**	**0.642**	0.067	**9.624**	<0.001 ***	0.511	0.773	0.283	3.528
	**Marital Status (Ref: Married)**									
	Single		−0.256	1.395	−0.184	0.854	−2.996	2.483	0.439	2.278
	**Educational Status (Ref: Postgraduate)**									
	Literate		**27.595**	2.461	**11.213**	<0.001 ***	22.761	32.428	0.364	2.750
	Primary Education		**20.527**	2.699	**7.605**	<0.001 ***	15.226	25.828	0.407	2.458
	High School		**12.616**	1.954	**6.458**	<0.001 ***	8.779	16.453	0.399	2.506
	University		1.861	1.450	1.284	0.200	−0.986	4.709	0.400	2.499
	**Income Status (Ref: Income Greater Than Expenses)**									
	Income Lower Than Expenses		1.440	1.376	1.047	0.296	−1.262	4.143	0.495	2.020
	Income Equal to Expenses		−0.655	1.332	−0.492	0.623	−3.271	1.961	0.645	1.549
	**Smoking Status (Ref: Yes)**									
	No		**−3.185**	1.281	**−2.487**	0.013 *	−5.701	−0.669	0.692	1.446
	Quit		0.956	3.356	0.285	0.776	−5.636	7.549	0.607	1.646
	**Alcohol Consumption Status (Ref: Yes)**									
	No		−3.453	2.449	−1.410	0.159	−8.263	1.357	0.590	1.695
	Quit		−5.513	4.542	−1.214	0.225	−14.435	3.409	0.480	2.084
	**Meal Skipping Status (Ref: Yes)**									
	No		−0.196	1.018	−0.193	0.847	−2.195	1.803	0.933	1.072
	**Daily Water Intake (Ref: ≥2 L/day)**									
	0 ≤ 1.5 L/day		0.857	1.449	0.591	0.555	−1.989	3.703	0.720	1.390
	1.5 ≤ 2 L/day		0.946	1.074	0.881	0.379	−1.163	3.056	0.731	1.368
	**BMI**		0.447	0.247	1.811	0.071	−0.038	0.931	0.231	4.322
	**Waist-to-Hip Ratio**		−0.179	14.058	−0.013	0.990	−27.790	27.432	0.224	4.464
	**Total DASH**		−0.034	0.076	−0.439	0.661	−0.184	0.116	0.754	1.327
	**Total MEDAS**		−0.036	0.298	−0.120	0.905	−0.621	0.550	0.677	1.476
	**TMT Total**	(Constant)	**39.527**	13.823	**2.859**	0.004 **	12.377	66.677		
		**Sex (Ref: Female)**								
		Male	2.506	1.877	1.336	0.182	−1.179	6.192	0.462	2.166
		**Age**	**0.954**	0.092	**10.391**	<0.001 ***	0.774	1.135	0.283	3.528
	**Marital Status (Ref: Married)**									
	Single		−0.278	1.921	−0.145	0.885	−4.051	3.495	0.439	2.278
	**Educational Status (Ref: Postgraduate)**									
	Literate		**40.754**	3.390	**12.023**	<0.001 ***	34.097	47.411	0.364	2.750
	Primary Education		**30.255**	3.718	**8.138**	<0.001 ***	22.953	37.556	0.407	2.458
	High School		**18.522**	2.691	**6.883**	<0.001 ***	13.237	23.807	0.399	2.506
	University		3.197	1.997	1.601	0.110	−0.726	7.119	0.400	2.499
	**Income Status (Ref: Income Greater Than Expenses)**									
	Income Lower Than Expenses		2.383	1.895	1.257	0.209	−1.339	6.105	0.495	2.020
	Income Equal to Expenses		−0.572	1.834	−0.312	0.755	−4.175	3.031	0.645	1.549
	**Smoking Status (Ref: Yes)**									
	No		**−4.601**	1.764	**−2.608**	0.009 **	−8.066	−1.136	0.692	1.446
	Quit		1.208	4.623	0.261	0.794	−7.872	10.288	0.607	1.646
	**Alcohol Consumption Status (Ref: Yes)**									
	No		−5.225	3.373	−1.549	0.122	−11.850	1.400	0.590	1.695
	Quit		−7.666	6.256	−1.225	0.221	−19.954	4.622	0.480	2.084
	**Meal Skipping Status (Ref: Yes)**									
	No		−0.269	1.402	−0.192	0.848	−3.023	2.485	0.933	1.072
	**Daily Water Intake (Ref: ≥2 L/day)**									
	0 ≤ 1.5 L/day		1.309	1.996	0.656	0.512	−2.611	5.228	0.720	1.390
	1.5 ≤ 2 L/day		1.457	1.479	0.985	0.325	−1.449	4.363	0.731	1.368
	**BMI**		0.589	0.340	1.734	0.083	−0.078	1.256	0.231	4.322
	**Waist-to-Hip Ratio**		10.875	19.362	0.562	0.575	−27.155	48.904	0.224	4.464
	**Total DASH**		−0.031	0.105	−0.295	0.768	−0.238	0.176	0.754	1.327
	**Total MEDAS**		−0.027	0.410	−0.067	0.947	−0.833	0.779	0.677	1.476

Model-1: Adj. R^2^ = 0.777; R2 = 0.786; F = 91.835; *p* < 0.001; Model-2: Adj. R2 = 0.764; R2 = 0.773; F = 85.356; *p* < 0.001; Model-3: Adj. R2 = 0.693; R2 = 0.704; F = 59.680; *p* < 0.001; Model-4: Adj. R2 = 0.723; R2 = 0.734; F = 69.086; *p* < 0.001. Model 1: Total Recall (Oktem-VMPT); Model 2: TMT-A (Trail Making Test-A); Model 3: TMT-B (Trail Making Test-B); Model 4: Total TMT (Total Trail Making Test). BMI: Body Mass Index; TMT-A: Processing Speed Based on Visual Scanning Ability; TMT-B: Ability to Shift Set Between Stimulus Sets and Follow Sequencing; SE: Standard Error. * *p* < 0.05; ** *p* < 0.01; *** *p* < 0.001.

**Table 5:** Correlation analysis of the relationships between DASH and MEDAS scores and the Oktem-VMPT and TMT subcomponents according to participants’ sex.

	Male				Female				Total			
	DASH Total		MEDAS Total		DASH Total		MEDAS Total		DASH Total		MEDAS Total	
	s	*p*	s	*p*	s	*p*	s	*p*	s	*p*	s	*p*
Oktem-VMPT Subscales												
Immediate Memory	0.164	0.009	0.164	0.008	0.209 *	0.000 *	−0.041	0.448	0.198 *	0.000 *	0.083	0.043
Learning Score	0.030	0.628	0.110	0.078	0.156	0.004	0.022	0.687	0.121	0.003	0.119	0.004
Reaching Criterion	0.051	0.412	0.100	0.110	0.143	0.008	0.128	0.017	0.124	0.002	0.179 *	0.000 *
Highest Learning	0.051	0.412	0.100	0.100	0.143	0.008	0.128	0.017	0.124	0.002	0.179 *	0.000 *
Learning Error Score	−0.243 *	0.000 *	−0.218 *	0.000 *	−0.219 *	0.000 *	−0.014	0.792	−0.235 *	0.000 *	−0.130 *	0.001 *
Spontaneous Recall	0.126	0.043	0.156	0.012	0.274 *	0.000 *	−0.002	0.971	0.228 *	0.000 *	0.131 *	0.001 *
Recognition	0.025	0.685	0.062	0.323	0.145	0.007	0.123	0.023	0.108	0.008	0.152 *	0.000 *
Total Recall	0.099	0.113	0.138	0.027	0.242 *	0.000 *	0.019	0.722	0.198 *	0.000 *	0.140 *	0.001 *
LTM Error Score	−0.219 *	0.000 *	−0.149	0.017	−0.204 *	0.000 *	0.038	0.478	−0.213 *	0.000 *	−0.039	0.337
TMT-A	−0.156	0.012	−0.125	0.046	−0.254 *	0.000 *	0.007	0.892	−0.225 *	0.000 *	−0.093	0.023
TMT-B	−0.146	0.019	−0.140	0.025	−0.229 *	0.000 *	0.019	0.727	−0.209 *	0.000 *	−0.093	0.023
TMT Total	−0.152	0.015	−0.133	0.033	−0.242 *	0.000 *	0.013	0.812	−0.217 *	0.000 *	−0.094	0.022

TMT-A: Processing Speed Based on Visual Scanning Ability; TMT-B: Ability to Shift Set Between Stimulus Sets and Follow Sequencing; LTM: Long-Term Memory; s: Spearman’s Rank-Order Correlation Coefficient; Bonferroni-corrected *p* = 0.002; * *p* < 0.002.

## Data Availability

The data used for the analyses in this study can be made available upon a reasonable request to the corresponding author and will require approval from the principal investigator’s and site collaborator’s Institutional Review Boards. It will only be shared if it is ethically correct to do so, where this does not violate the protection of human subjects, or other valid ethical, privacy, or security concerns.

## Abbreviations

The following abbreviations are used in this manuscript:

MEDAS	Mediterranean Diet Adherence Screener (MEDAS)
DASH	Diet Quality Scale
Oktem-VMPT	Oktem Verbal Memory Processes Test
TMT	Trail Making Test
DASH-Q	DASH Diet Quality Scale
